# Improvement of vibrodamping properties of polyvinyl acetate–graphite composites by electron beam processing of the filler

**DOI:** 10.1186/s40064-016-3193-2

**Published:** 2016-09-13

**Authors:** Sergey V. Mjakin, Maxim M. Sychov, Nadezhda B. Sheiko, Larisa L. Ezhenkova, Alexander G. Rodionov, Inna V. Vasiljeva

**Affiliations:** 1 Saint-Petersburg State Institute of Technology (Technical University), 26 Moskovsky pr., St. Petersburg, Russia 190013; 2 Engineering Technology Center RADIANT, Ltd., 50 Dibunovskaya str., St. Petersburg, Russia 197183

**Keywords:** vibrodamping, Polyvinyl acetate, Graphite, Surface, Functional groups

## Abstract

The effect of electron beam processing (energy 900 keV, absorbed dose in the range from 25 to 600 kGy) of graphite upon the efficiency of its use as a filler in polyvinyl acetate (PVA) based vibrodamping composites is studied. Graphite treatment at optimal doses above 200 kGy is found to provide a significant increase of damping loss factor for these composites at ambient and especially at elevated temperatures. The observed improvement of vibrodamping properties correlates with the increase in the content of Broensted centers (hydroxyl groups) on modified graphite surface probably due to the additional bonding of the filler particles with each other and PVA binder.

## Background

Polymer based composites involving various dispersed, fibrous and layered fillers are one of the main types of modern vibration damping materials (VPM). Their vibrodamping properties are determined by viscoelastic state of the polymer binder and interaction of its macromolecules with the filler to form a network of bonds affording an additional effective absorption of mechanical vibrations (Thomas et al. 2013). These features determine a high importance of the study of processes at the polymer–filler boundary interface responsible for a strong dependence of VPM properties upon the filler content, dispersity, surface energy and other factors. While the polymer binder is mostly responsible for the vibration damping properties, the addition of dispersed functional fillers is required to provide the target exploration performances of the composites and reduce their cost.

The filler and binder phases in polymer based composites are separated by interphase layers formed as a result of polymer–filler interaction via the adsorption mechanism due to a high surface energy and surface area of the filler in combination with the presence of specific active centers on dispersed filler particles (Thomas et al. 2013; Denisyuk and Fokina 2010). Therefore, the adjustment of the filler surface functional composition is an important approach to the enhancement of their vibrodamping properties.

Modern VPM are often based on polyvinyl acetate (PVA) as a polymer binder due to its high damping loss factor, good thermoplastic properties, thermal stability, availability and non-toxicity in combination with carbon materials as inert and highly available dispersed fillers. Particularly, a mastic with high vibrodamping properties on the basis of PVA dispersion and graphite as one of the most efficient fillers was suggested in (2002). A possible approach to the further enhancement of vibrodamping performances for such composites is based on the improvement of the filler–binder compatibility due to a specific functionalization of the filler surface in order to strengthen its interaction with the polymer.

In our earlier studies (Alekseev et al. 2006; Myakin et al. 2011) it was shown that such important performances of composites as homogeneity, permittivity, etc. can be improved by a specific modification of the dispersed filler surface in order to increase the amount of certain active centers responsible for acid–base interactions with the polymer binder. Particularly, properties of polyvinyl alcohol cyanoethyl ester based composites were found to strongly correlate with the content of Broensted base centers on BaTiO_3_ filler surface due to their interaction with acid groups of the polymer.

In Vasiljeva et al. (2002, 2006), Shmykov et al. (2009), Mjakin et al. (2009) electron beam (EB) modification of the surface of various solids was demonstrated to provide the formation of reactive hydroxyls with adjustable acidity affording the enhancement of support-functional layer and binder–filler interactions. In Banhart (1999) EB processing was described as an effective approach to induce adjustable structural transformations on the surface of various carbon materials, including the formation of radiation defects in graphite. In this study the considered approach was used to improve vibrodamping performances of PVA–graphite compositions.

## Methods

The studied vibrodamping composite involved the following components:

- 40 % PVA dispersion (50 % aqueous dispersion of PVA with the average particle size 1–3 μm, density 1.17 g/cm^3^ and dynamic viscosity 2.0–5.0 Pa s);
- 40 wt% graphite (crystalline, foundry, GL-2 brand, ash content no higher than 18 %) as the main filler;
- 7 wt% dibutyl phthalate as a plasticizer;
- 13 wt% nepheline fire retardant.

PVA composition was studied by 500 MHz ^1^H NMR- and ^13^C-NMR spectroscopy using a Bruker AM-500 spectrometer with a 5 mm carbon-proton detector at ambient temperature in deuterated dimethylsulfoxide (CD_3_)_2_SO (DMSO-d_6_), residual proton signal at 2.50 ppm, carbon signal at 39.52 ppm. In order to avoid the distortion of the integral intensities for the analyzed signals the spectra were measured with a relaxation delay within 20 s with the proton suppression switched off.

EB processing of graphite was carried out using a resonance-transforming electron accelerator RTE-1V with the energy 900 keV, current 1 mA and absorbed dose in the range from 25 to 600 kGy. The samples were processed and stored in air atmosphere under ambient conditions. The absorbed dose was determined by the processing time and checked by spectrophotometric measurement of optical density for copolymer films CDP-F2 (containing a phenazine dye) at λ = 512 nm according to the standard calibrating tables and plots for optical density as function of the absorbed dose. In order to provide a uniform treatment, graphite samples were placed under the accelerator outlet window as a layer of about 1 mm thickness less than the penetration depth of accelerated electrons.

The functionality of graphite surface were studied using the selective adsorption of a series of acid–base indicators with different intrinsic pK_a_ values in the range from −5 to 15 from their aqueous solutions on the surface centers with the corresponding pK_a_ values according to the approach generally described in Tanabe (1971), Nechiporenko (1995) and experimental procedure described in detail in Vasiljeva et al. (2002, 2006), Shmykov et al. (2009), Mjakin et al. (2009). Due to the dissociation of indicator molecules with specific pK_a_ constants, their dissociated moieties tend to selectively adsorb on certain surface centers (also undergoing dissociation in water with similar pKa values) (Tanabe 1971; Nechiporenko 1995; Tyurin Ju 2001) to substitute for such dissociated species as chemisorbed or physically adsorbed water, hydroxyls, protons, etc.) in accordance with water dissociation constant К_w_ = pH + pOH = pK_a_ + pK_b_ = 14. Particularly, indicators with the lowest (usually negative) pK_a_ values are selectively adsorbed on Lewis basic centers which possess free electron pair and are capable to interact with protons. With the increase of intrinsic pK_a_ values of the indicators their adsorption takes place on the corresponding Broensted acidic sites (pK_a_ ~ 0…7, surface –OH groups capable of proton abstraction), Broensted basic centers (pK_a_ ~ 7…14, surface –OH groups with the tendency to split off hydroxyl ion) and Lewis acidic centers (pK_a_ higher than ~14, by electron accepting atoms or ions). The content of the analyzed adsorption centers Q (μmol/g) was determined according to the changes in optical density of indicator solutions using a spectrophotometer SF-46 (LOMO, St. Petersburg, Russia).

Vibrodamping properties of the obtained materials were characterized using the Test Oberst procedure at frequencies 200–800 Hz and temperatures 17–100 °C. The resonance peak width vs frequency was measured and damping loss factor (k) was determined at a constant applied force for bending vibrations of console steel rod samples with the length 230 ± 0.1 mm, width 8 ± 0.1 mm and thickness 1.5 ± 0.1 mm damped with a double layer of the studied material. Each composite was studied by performing the described measurements for 5 rods followed by the elimination of the lowest and highest values and averaging with the acceptable difference between the measured values no more than ±0.02.

## Results and discussion

The characterization of graphite surface indicates a diversity of functional groups (Table 1) probably determined by the presence of carbon in a non-oxidized (typical for graphite) and oxidized states (probably relating to almost neutral centers with pK_a_ 6.4 and Lewis acidic sites with pK_a_ 14.2 correspondingly) as well as oxygen-containing groups such as C=O (Lewis basic sites with pK_a_ < 0) and hydroxyls representing Broensted acidic (pK_a_ 2.5) and basic (pK_a_ 8.8) centers (Ostrovsky and Virgiljev 1986).

**Table 1:** Content of various centers on graphite surface as a function of absorbed dose at electron beam processing

Absorbed dose (kGy)	Content of surface centers with different pK_a_ values (μmol/g)					
	pK_a_ −4.4	pK_a_ 2.5	pK_a_ 6.4	pK_a_ 8.8	pK_a_ 14.2	10.5
0	26.3	2.1	7.1	0.7	25,1	0.92
25	6.2	1.1	0.2	1.8	16.0	0.13
50	6.0	5.4	0.1	1.3	4.0	0.44
75	31.5	1.5	2.0	2.1	17.2	0.58
100	1.8	0.3	4.6	3.8	1.1	0.59
150	1.9	0.7	2.9	1.6	23.1	0.66
200	7.0	4.4	2.8	3.0	29.7	0.04
300	7.1	4.3	3.2	2.6	5.7	0.95
400	0.3	5.6	6.2	0.3	34.8	1.16
600	3.8	2.4	7.2	0.8	1.4	0.64

The observed significant changes in the content of the considered functional groups can be determined by the alternating processes of partial dehydration (dehydroxylation) due to the radiation heating and hydroxylation due to the interaction with H^·^ and OH^·^ radicals formed at the radiolysis of adsorbed water molecules. In a series of our previous studies (Vasiljeva et al. 2002, 2006; Shmykov et al. 2009; Mjakin et al. 2009) similar functional transformations were observed on the surface of various materials including dispersed oxides, ceramics, polymers, etc.

**Fig. 1:**
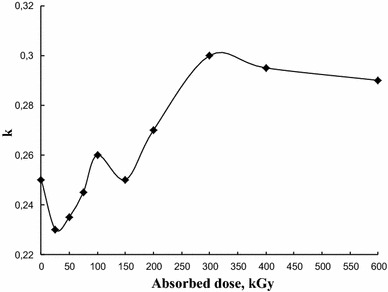
Mechanical loss factor of PVA–graphite composites at 24 °C as a function of absorbed dose at EB processing of graphite filler

EB pretreatment of graphite filler samples at absorbed doses above 200 kGy resulted in a considerable increase in the damping loss factor for the corresponding composites at ambient conditions (temperature 24 °C) (Fig. 1). The highest growth by about 20 % (from 0.25 to 0.3) relating to the use of non-modified graphite observed at 300 kGy is a prominent improvement for the studied material advancing it to the top level among various vibration damping composites (Chung 2001, 2003; Lakes 2002).

**Fig. 2:**
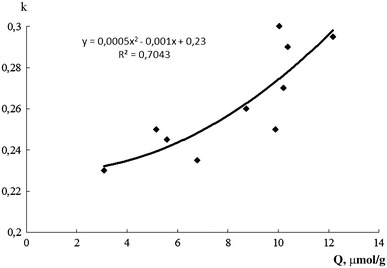
Mechanical loss factor of PVA–graphite composites at 24 °C as a function of the content of adsorption centers with pKa 2.5, 6.4 and 8.8 on the graphite filler surface

Furthermore, damping loss factor of the studied composites is found to have a good correlation with the total concentration of Broensted centers on graphite filler surface best fitted with a parabolic equation (Fig. 2) in the considered range of these centers content and approximated to k ~ 0.23 for the material free of this centers (corresponding to the non-modified graphite).

**Fig. 3:**
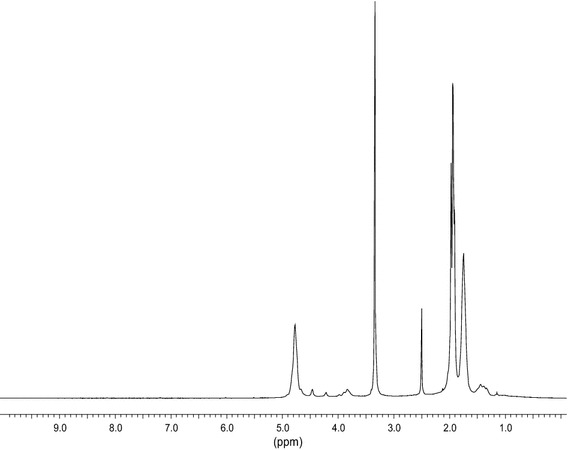
500 MHz ^1^H NMR spectrum of PVA dispersion. 4.77 ppm—chain methine groups in acetate units; 4.67, 4.46, 4.21 ppm—hydroxyl groups in alcohol units; 4.08–3.64 ppm—chain methine groups in alcohol units; 3.34 ppm—water; 2.50 ppm—solvent (DMSO); 2.16–1.57 ppm—chain methylene and side methine groups in acetate units; 1.57–1.21 ppm—chain methylene groups in alcohol units. Molar content of alcohol units [OH] was calculated from the data for low- and high-field regions as $[{\text{OH}}] = \frac{{{\text{I}}(4.08 - 3.64)}}{{{\text{I}}(5.1 - 4.1)}}$ and $[{\text{OH}}] = \frac{{{\text{I}}(1.57 - 1.21)/2}}{{{\text{I}}(1.57 - 1.21)/2 + {\text{I}}(2.16 - 1.57)/5}}$ giving the result of [OH] about 10 %

**Fig. 4:**
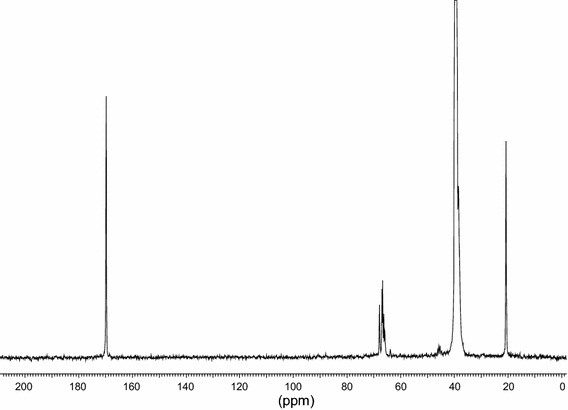
^13^C NMR spectrum of PVA dispersion. 169.71 ppm—carbonyl side groups in side chains of vinyl acetate units; 68.4–63.3 ppm—chain methine groups in acetate and alcohol units; 46.5–44.0 ppm—chain methylene groups in alcohol units; 39.52 ppm—solvent; 42.3–35.0 ppm—chain methylene groups in acetate units (overlapped with the solvent peak); 20.73 ppm—methyl groups in side chains of vinyl acetate units. Molar content of alcohol units $[ {\text{OH]}}$ was calculated as $[{\text{OH}}] = \frac{{{\text{I}}(46.5 - 44.0)}}{{{\text{I}}(46.5 - 44.0) + {\text{I}}({\text{Ac}})}}$ where I(46.5−44.0)] is integral intensity of peaks corresponding to alcohol units and I(Ac) is the intensity of peaks at 169.71 or 20.73 ppm corresponding to carbonyl or methyl groups respectively, giving the result of [OH] about 12 % in both cases

**Fig. 5:**
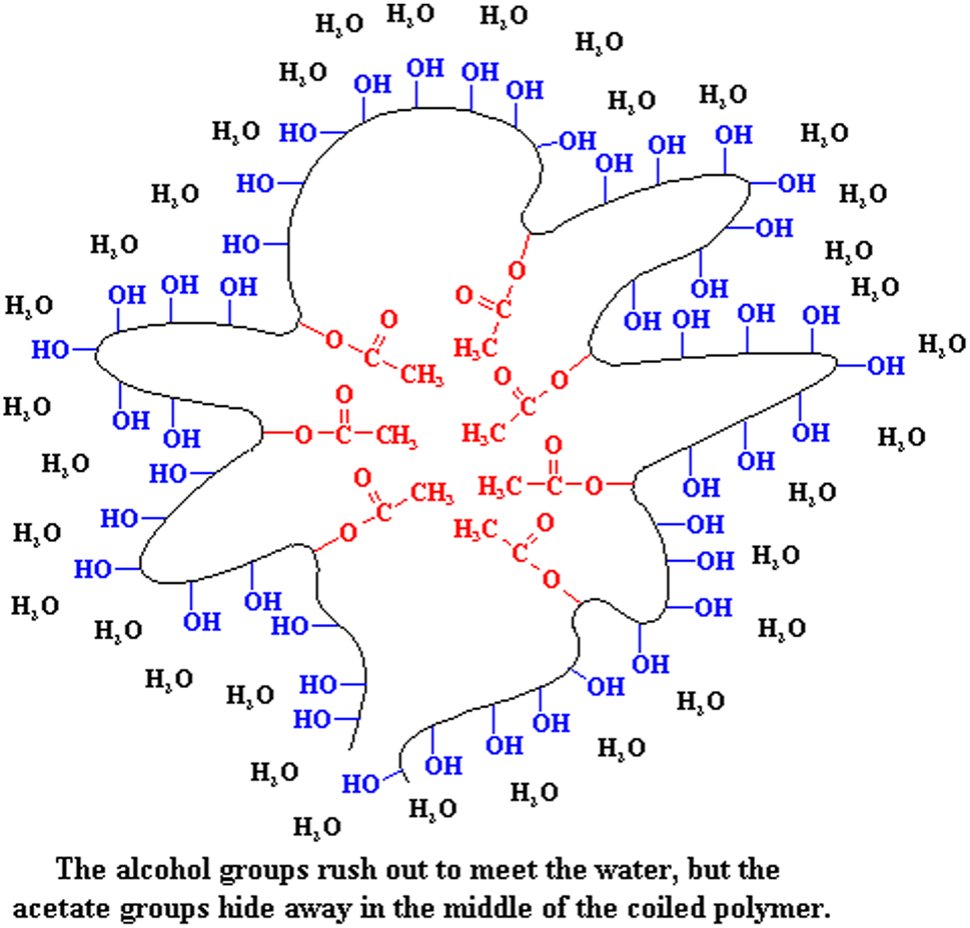
PVA coil structure

This correlation suggests a possible mechanism of the observed effect relating to the vibration energy dissipation on the network of bonds resulting from a partial interfacial condensation of hydroxyl groups of the filler and PVA. A relatively high content of hydroxyls in the applied PVA dispersion is confirmed by both ^1^H- and ^13^C-NMR data indicating a significant (10–12 wt%) content of polyvinyl alcohol (Figs. 3, 4). Since PVA surface is largely occupied by hydroxyls (especially in aqueous dispersions used for obtaining the considered composites; http://chemsrv1.uwsp.edu/macrogcss/pva.html) (Fig. 5) it is the content of OH groups on the graphite filler surface that predominantly determines and limits the interfacial interactions in the considered composites.

The characterization of vibrodamping properties at elevated temperatures (35–38 °C) indicates a significantly more promising increase of damping loss factor due to EB processing of graphite filler. In this case measurements performed only for composites containing graphite treated at relatively high absorbed doses showed a growth of damping loss factor from 1.5 to about 2.5 times for 300 and 600 kGy correspondingly compared with the composites with non-modified graphite (Table 2). This behavior confirms the formation of an additional network of bonds preventing from heat motion of macromolecular segments at elevated temperatures.

**Table 2:** Mechanical loss factor of PVA–graphite composites at 35–38 °C as a function of absorbed dose at EB processing of graphite filler

Absorbed dose (kGy)	k
0	<0.1
300	0.13
400	0.17
600	0.23

## Conclusions

Electron beam treatment (energy 900 keV, absorbed dose in the range 200–600 kGy) of graphite subsequently used as a filler in PVA based composites provides a significant increase in the damping loss factor of these materials at ambient and especially at elevated (35–38 °C) temperatures. The observed improvement of vibrodamping properties correlates with the increase in the content of hydroxyls on the modified graphite surface enhancing the filler–polymer and filler–filler interaction to form a network of bonds absorbing the vibration energy.

## References

[CR4] Alekseev SA (2006). Effect of donor–acceptor sites at a barium titanate surface on the properties of composites based on cyanoethyl polyvinyl alcohol. Russ J Appl Chem.

[CR3] Bakutkin Ju I et al. (2002) Patent RU 2,181,739

[CR5] Banhart F (1999). Irradiation effects in carbon nanostructures. Rep Prog Phys.

[CR6] Chung DDL (2001). Materials for vibration dumping. J Mater Sci.

[CR7] Chung DDL (2003). Structural composite materials tailored for damping. J Alloy Compd.

[CR1] Denisyuk I, Fokina M (2010) A review of high nanoparticles concentration composites: semiconductor and high refractive index materials, nanocrystals. In: Masuda Y (Ed.), ISBN: 978-953-307-126-8, InTech. http://www.intechopen.com/books/nanocrystals/high-nanoparticlesconcentration-composites-semiconductor-and-high-refractive-index-materials

[CR8] Lakes RS (2002). High damping composite materials: effect of structural hierarchy. J Compos Mater.

[CR9] Mjakin SV, Sychov MM, Vasiljeva IV (2009). Electron beam modification of solids: mechanisms, common features and promising applications.

[CR10] Myakin SV (2011). Effect of the modification of barium titanate on the permittivity of its composites with cyanoethyl ester of polyvinyl alcohol. Glass Phys Chem.

[CR11] Nechiporenko AP (1995) Acid-base properties of the surface of solid oxides and chalcogenides. Thesis on Dr. Sci. degree/St-Petersburg State Institute of Technology (Technical University) **(in Russian)**

[CR12] Ostrovsky VS, Virgiljev VI (1986). Iskusstvenny graphit (Artificial graphite).

[CR13] Shmykov AY (2009). Electron beam initiated grafting of methacryloxypropyl-trimethoxysilane to fused silica. Appl Surf Sci.

[CR14] Tanabe K (1971). Solid acids and bases.

[CR2] Thomas S (2013). Polymer composites, nanocomposites.

[CR15] Tyurin Ju I (2001). Hemovozbuzhdenie poverhnosti tverdyh tel (Chemical excitation of the surface of solids).

[CR16] Vasiljeva IV (2002). Electron beam modification of the surface of oxide materials (SiO_2_, BaTiO_3_). Russ J Phys Chem.

[CR17] Vasiljeva IV (2006). Electron beam induced modification of poly(ethylene terephthalate) films. Appl Surf Sci.

